# Belatacept Removal by Plasmapheresis: Dose Adjustments and Clinical Recommendations

**DOI:** 10.1097/TXD.0000000000001677

**Published:** 2024-07-05

**Authors:** Nicole K. Wilson, Simon Tremblay, Adele R. Shields, E. Steve Woodle, Rita R. Alloway, Alexander A. Vinks, Bradley Miyagawa, Tomoyuki Mizuno

**Affiliations:** 1 Department of Surgery, University of Cincinnati, Cincinnati, OH.; 2 Department of Pharmacy, Baylor University Medical Center at Dallas, Dallas, TX.; 3 Veloxis Pharmaceuticals, Inc, Cary, NC.; 4 Department of Surgery, The Christ Hospital, Cincinnati, OH.; 5 Division of Clinical Pharmacology, Cincinnati Children’s Hospital Medical Center, Cincinnati, OH.; 6 Department of Pediatrics, College of Medicine, University of Cincinnati, Cincinnati, OH.; 7 Division of Pharmaceutical Sciences, James L. Winkle College of Pharmacy, University of Cincinnati, Cincinnati, OH.

Belatacept, a fusion protein immunosuppressant, may be removed by plasmapheresis (PP) in a clinically significant amount. Scenarios arise requiring concurrent PP in patients maintained on belatacept; however, clinical guidance for dosing remains an unmet need. We propose a supplemental dosing strategy of belatacept in the setting of PP.

Kidney transplant (KT) recipients included received de novo belatacept as part of the BEST (Belatacept Early Steroid Withdrawal Trial) randomized controlled trial at the University of Cincinnati Medical Center or The Christ Hospital.^1,2^ During the trial, a belatacept supplemental dosing strategy was established for KT undergoing PP for cause. To evaluate this strategy, belatacept concentration measurements were retrospectively analyzed. Serum samples collected to test for donor-specific antibodies for cause and at prespecified time points were used for belatacept concentration measurement.^3^ Belatacept exposure was compared in KT recipients who did not receive supplemental belatacept and the subsequent KT recipients who were supplemented during PP.

A 50% supplemental dose was given after every 2 PP sessions regardless of time post-KT. During the first month post-KT, a full dose was given upon PP series completion. The next dose was scheduled 28 ± 3 d later, rather than the original schedule before initiation of PP. For recipients >1 mo post-KT, if a dose was previously scheduled <7 d after the final PP session, a full dose was given after completion of PP with the next dose rescheduled for 28 ± 3 d later. If a dose was previously scheduled >7 d after the final PP session, a 50% supplemental dose was given after the final PP session and the previously established dosing schedule was continued.

Belatacept individual pharmacokinetic profiles were generated with Bayesian estimation using MwPharm++ (Mediware, Prague, Czech Republic). A published population pharmacokinetic model was used as the Bayesian prior.^4^ Serum concentrations collected after PP events were compared with the model-based predicted profile.

Seven KT recipients underwent PP during the study period (median 4 sessions, range 1–4). Three PP series were performed within the first month post-KT. Three recipients did not receive supplemental belatacept doses after PP before the guidance was established.

Recipient demographics included 1 female and 6 males. Median age at the time of KT was 40 y (range 35–60 y). Median values for body mass index, height, and weight were 31.7 kg/m^2^ (range 20.0–36.5 kg/m^2^), 180.3 cm (range 152.4–188.0 cm), and 107.0 kg (range 46.5–114.3 kg), respectively.

**FIGURE 1.**
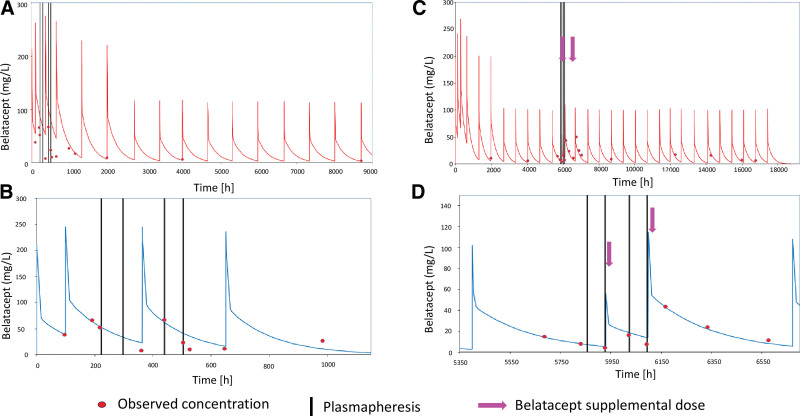
Belatacept pharmacokinetic profiles (A) Population and Bayesian-predicted belatacept concentration–time profiles (B) for 1 patient with no supplemental belatacept dose given after PP during the first month posttransplant. Population (C) and Bayesian-predicted belatacept concentration–time profiles (D) for 1 patient with empiric belatacept supplementation during and after PP >1 mo posttransplant. Bayesian estimations were performed using concentrations before the first PP.

Patients undergoing PP within the first month post-KT had lower-than-predicted belatacept concentrations, especially if supplemental belatacept doses were not given (Figure 1A). In the 3 recipients who received PP within the first month of belatacept initiation, 5 post-PP concentrations were available from 2 patients. The mean observed/predicted concentrations was 47.8% (range 19.4%–86.2%). The impact of PP was not as pronounced for patients who underwent PP >1 mo post-KT and who received supplemental dosing (Figure 1B).

Administering half of the standard belatacept dose after every second PP session may mitigate the impact of PP, particularly after the initial 10 mg/kg dosing phase. Further studies specifically investigating the use of belatacept in patients undergoing PP therapy are warranted to validate these findings.
